# Internalized Transphobia, Resilience, and Mental Health: Applying the Psychological Mediation Framework to Italian Transgender Individuals

**DOI:** 10.3390/ijerph15030508

**Published:** 2018-03-13

**Authors:** Cristiano Scandurra, Vincenzo Bochicchio, Anna Lisa Amodeo, Concetta Esposito, Paolo Valerio, Nelson Mauro Maldonato, Dario Bacchini, Roberto Vitelli

**Affiliations:** 1 Department of Neurosciences and Reproductive and Odontostomatological Sciences, University of Naples Federico II, 80131 Napoli, Italy; valerio@unina.it (P.V.); nelsonmauro.maldonato@unina.it (N.M.M.); rvitelli@unina.it (R.V.); 2 Department of Humanistic Studies, University of Calabria, 87036 Rende, Italy; vincenzo.bochicchio@unical.it; 3 Department of Humanistic Studies, University of Naples Federico II, 80133 Napoli, Italy; amodeo@unina.it (A.L.A.); concetta.esposito3@unina.it (C.E.); dario.bacchini@unina.it (D.B.)

**Keywords:** internalized transphobia, resilience, mental health, mediation, transgender

## Abstract

Transgender and gender nonconforming (TGNC) people are a highly-stigmatized population. For this reason, they might internalize society’s normative gender attitudes and develop negative mental health outcomes. As an extension of the minority stress model, the psychological mediation framework sheds light on psychological processes through which anti-transgender discrimination might affect mental health. Within this framework, the current study aimed at assessing in 149 TGNC Italian individuals the role of internalized transphobia as a mediator between anti-transgender discrimination and mental health, considering resilience as the individual-level coping mechanism buffering this relationship. The results suggest that both indicators of internalized transphobia (i.e., shame and alienation) mediate the relationship between anti-transgender discrimination and depression, while only alienation mediates the relationship between anti-transgender discrimination and anxiety. Furthermore, the results suggest that the indirect relation between anti-transgender discrimination and anxiety through alienation is conditional on low and moderate levels of resilience. Findings have important implications for clinical practice and psycho-social interventions to reduce stigma and stress caused by interpersonal and individual stigma.

## 1. Introduction

Transgender and gender nonconforming (TGNC) people are those whose gender identity is not fully aligned with the sex assigned at birth [1]. TGNC people are a highly stigmatized population facing systematic violence and oppression because of their gender nonconformity [2]. Anti-transgender discrimination leads to negative mental health outcomes, such as depression and anxiety [3,4]. Nevertheless, how psychological processes through which anti-transgender discrimination affects health and protective factors buffer this relation still remain under researched [5]. To this end, it seems urgent to understand potential mediators able to explain the relationship between anti-transgender discrimination and health outcomes.

One of the theoretical frameworks exploring the impact of stigmatizing processes on mental health of minority groups is the *minority stress theory* (MST) [6,7]. This theory posits that disparities originate within the stigmatizing social climate to which minority groups are exposed; this is why these groups are at high risk of developing negative health outcomes. MST was developed specifically with cisgender sexual minorities (i.e., lesbian, gay, and bisexual individuals), yet only recently it was empirically applied to the TGNC population [3,8,9], showing its potentialities in understanding TGNC experiences and capturing the challenges faced by this population. It has been widely demonstrated that experiencing violence and discrimination in high rates leads TGNC individuals to direct societal negative attitudes towards themselves and that, at the same time, resilience is exercised to contrast societal stigma nestled within a society which discriminates on the basis of gender identity [3,8]. Nevertheless, TGNC individuals experience unique health disparities due to their gender identity and we still need to increase our knowledge of their specific stressful experiences, as well as their unique adaptive strategies to face adversity.

Recent extensions of the MST—specifically, the *psychological mediation framework* (PMF) [10]—shed light on the role of group-specific processes (in particular, proximal stressors, such as internalized transphobia) and general psychological processes (e.g., emotion dysregulation) as mediators of the relationship between distal stressors (i.e., anti-transgender discrimination) and health. Furthermore, this framework called for research analyzing the individual- (e.g., resilience) and group-level coping mechanisms (e.g., involvement in collective action) able to buffer the relationship between distal/proximal stressors and health, also turning attention to a moderated mediation framework.

Within the PMF, the current study analyzed the role of the most proximal stressor (i.e., internalized transphobia) as a mediator between anti-transgender discrimination and mental health, considering resilience as the individual-level coping mechanism buffering this relationship. In the following paragraphs, we first provide an overview of research on minority stress in the TGNC population, with particular attention on anti-transgender discrimination, internalized transphobia, and resilience. Then, we present the PMF. Finally, since our study concentrates on Italy, we provide an overview of the social context for Italian TGNC people.

### 1.1. Minority Stress, Health, and Resilience in the TGNC Population

The MST assumes that sexual and gender minorities experience stress due to persistent social stigmatization. Within the context of the individual environmental circumstances, Meyer [7] conceptualizes distal and proximal stress processes. Distal processes are objective stressors that are independent of the individual because they operate beyond personal control. Yet, proximal stressors are dependent on the individual because they are linked to subjective feelings, thoughts, and actions; nevertheless, they are still embedded and connected to a broader social context that perpetuates negative views towards sexual and gender minority groups.

Regarding the most distal stressors (i.e., prejudice events), evidence indicates that TGNC people suffer from high rates of violence and discrimination [2,11,12]. For instance, in a sample of 350 TGNC people recruited in Virginia, Bradford et al. [2] found that 41% experienced anti-transgender discrimination, and that the predictive factors associated with anti-transgender discrimination were being female-to-male (FtM), low socio-economic status, belongingness to an ethnic minority group, lack of health insurance, transgender awareness at younger age, substance use, and low rates of family support and community connectedness.

The most proximal stressor, or rather internalized transphobia, has received less attention in the scientific community although recently some authors started to explore this minority stressor in the TGNC population [8,13,14]. Internalized transphobia can be defined as a discomfort with one’s own TGNC identity due to the internalization of society’s normative gender expectations [15]. Bockting [15] differentiates the vertical internalized transphobia, that is the expression of prejudice toward oneself—or in other words, the shame toward one’s own TGNC identity—from the horizontal internalized transphobia, that is to say, the alienating feelings towards other TGNC people. In the transgender identity development model, Lev [16] states that TGNC individuals might experience shame and self-hatred in their initial stage of transgender identity, when they become aware of living an incongruence between gender identity and gender assigned at birth. Persistence of these feelings after this stage should be read as a sign of internalized transphobia [17]. However, even if many TGNC individuals are able to access social support and benefit from community connectedness, many others may experience alienating feelings towards other TGNC people because of the accumulation of stressors in the social contexts where they live [18], which tend to be strongly discriminatory.

Both anti-transgender discrimination and internalized transphobia lead to negative health outcomes. For instance, considering anti-transgender discrimination, Bockting et al. [3] found a positive association between social stigma and depression, anxiety, and somatization. Instead, with respect to internalized transphobia, Perez-Brumer et al. [19] reported that internalized transphobia increases the likelihood of suicide attempts. Specifically, shame towards one’s own TGNC identity resulted to be positively associated with perceived stress, depressive symptoms, and social anxiety symptoms [9], while alienation towards other TGNC people resulted in being positively associated with perceived stress, depressive symptoms, and anxiety [17].

Within the MST, there is evidence that TGNC individuals use adaptive strategies to buffer the effect of anti-transgender discrimination and internalized transphobia on their health [20,21,22,23]. Among these factors, resilience undoubtedly represents one of the main factors to successfully overcome negative and adverse life conditions, while promoting social adjustment [24,25]. Indeed, resilience represents a personal adaptive strategy that individuals may use to buffer the effects of stress on health, involving the adaptation to risk factors and the capability of “bouncing back” from adversity [25,26,27]. Resilience also represents the individual’s ability to negotiate with social contexts, generating a greater access to resources [28]. Evidence shows that this is also true for TGNC people. For instance, Singh et al. [21,22] qualitatively explored resilience within the TGNC population, finding that resilience involves both individual traits, such as an evolving a self-generated definition of self, embracing self-worth or awareness of oppression, and social characteristics, such as the connectedness to TGNC communities. Both individual traits and social characteristics are effective in decreasing negative outcomes of both distal and proximal stressors.

### 1.2. The Psychological Mediation Framework within the TGNC Population

Starting from a clinical view of stress, PMF was postulated as an extension of the MST in order to understand psychological pathways that link stigma-related stressors to negative mental health outcomes [10]. As reported by Hatzenbuehler [10], where MST posits that stress mediates the relationship between social structure/status and mental health, PMF postulates that stress is an initial starting point that leads to negative mental health outcomes through psychological mediators. Psychological mediators are both group-specific processes (in particular, the proximal stressors) and general psychological processes (such as emotion dysregulation or interpersonal problems). At the same time, this framework considers also the individual- (e.g., resilience) and group-level (e.g., group identification) coping mechanisms as specific dimensions buffering the effect of stressors on mental health, thus postulating a moderated mediation framework. Therefore, the MST postulates that both distal and proximal stressors predict mental health and coping mechanisms (e.g., resilience) buffer the effects of stigma on health. Yet, in the PMF distal stressors predict negative mental health outcomes, while proximal stressors mediate the relationship between distal stressors and health; instead, coping mechanisms buffer the relationship between both distal and proximal stressors and health.

Recent works have supported the PMF within sexual minority populations, including lesbian, gay, and bisexual cisgender people [29,30]. On the contrary, only a few studies have applied the PMF to the TGNC population.

The first study to apply the PMF to the TGNC population, also considering individual and group coping strategies as moderators, was conducted by Breslow et al. [5]. Within a sample of 552 TGNC adults, these scholars [5] found that stigma awareness, not internalized transphobia, mediated the relationship between anti-transgender discrimination and psychological distress. Likewise, they found that collective action, and not resilience, moderated the relationship between internalized transphobia and psychological distress. Thus, Breslow et al. [5] confirmed the hypothesis that a proximal stressor (i.e., stigma awareness) mediates the relationship between a distal stressor (i.e., anti-transgender discrimination) and health (i.e., psychological distress). Similarly, they confirmed that a group-level coping strategy (i.e., collective action) buffers the effect of proximal stressors on health. Despite this, this study failed in finding any significance inasfar as the role of internalized transphobia as a mediator between anti-transgender discrimination and psychological distress and resilience as a moderator between minority stressors and psychological distress. Breslow et al. [5] did not consider any general psychological process in their analyses. 

Another study that did not specifically apply the PMF to the TGNC population, but used many variables and relationships common to this framework, was the one conducted by Testa et al. [31]. Within a sample of 816 TGNC adults, and through the lens of the interpersonal-psychological theory of suicide, Testa et al. [31] found an indirect effect of prejudice events on suicide ideation via all proximal stressors. Furthermore, authors found evidence that internalized transphobia and negative expectations were associated with suicide ideation through thwarted belongingness and perceived burdensomeness.

A third study that specifically applied the PMF to TGNC population was that by Timmins et al. [32]. Authors found that all minority stressors (i.e., prejudice events, expectations of rejection, and internalized transphobia) were associated with psychological distress; they also found that rumination, used as a dimension measuring general psychological processes (i.e., emotion dysregulation) accounted to some extent for these relationships.

Finally, a fourth study applying the PMF to 201 transgender veterans explored the influence of distal and proximal minority stressors experienced during and after military service on suicide ideation [33]. Authors found that distal stressors (i.e., past-year anti-transgender discrimination and rejection) indirectly predicted suicide ideation through feelings of shame related to gender identity (i.e., proximal stressor).

Summarizing, actual scientific literature reports evidence that both group-specific processes [5,31,33] and general psychological processes [32] mediate the relationship between external stressors and negative mental health outcomes, and that group-level coping mechanisms [5] buffer the effect of stressors on mental health. Notwithstanding that, no previous studies took into account the internal differentiation of internalized transphobia while analyzing its vertical and horizontal nature. Furthermore, no previous studies homogeneously demonstrated that internalized transphobia would act as a mediator between distal stressors and health and that, at the same time, individual-level coping mechanisms (e.g., resilience) would buffer this relationship, finding evidence for this specific moderated-mediation model. As previously mentioned, Breslow et al. [5] failed in demonstrating this hypothesis and this led us to replicate the model containing internalized transphobia as a mediator in the current study, using a measure specifically created to assess horizontal and vertical internalized transphobia in the TGNC population (see the Section 2.3). Indeed, to assess internalized transphobia, Breslow et al. [5] used the four-item Private subscale of the Collective Self-Esteem Scale (CSES) [34], adapting it to the TGNC population. The items of the CSES—e.g., “I often regret that I belong to my gender identity group”—seem closer to the concept of horizontal internalized transphobia than the vertical one, because they ask about how people feel and perceive belonging to social groups. However, notwithstanding some similarities with the horizontal internalized transphobia, a measure not specifically created for the TGNC population might have influenced the results.

### 1.3. Stigma and Health in the Italian TGNC Population

The Italian context does not seem to be highly supportive of TGNC people [35,36,37,38,39]. Indeed, Italy is still lacking in anti-discrimination policies towards the TGNC population; on the basis of studies that focus on the healthy effect that inclusive social policies have on well-being of minority groups [19], this represents a serious health risk. 

Unfortunately, very few studies have focused on minority stress in Italian TGNC people. The only study [8] that thoroughly applied the MST to the Italian TGNC population found that prejudice events and internalized stigma were associated with negative mental health outcomes, specifically with depression, anxiety, and suicide ideation. Furthermore, support from family and resilience buffered the effect of prejudice events on health. Specifically, family support protected against anxiety, as resilience protected against depression and suicide ideation. No previous study tested the PMF, or part of it, within a sample of Italian TGNC people.

Additionally, a study by Amodeo et al. [40] presented findings from an empowerment-based group training program for a small group of TGNC youths who experienced anti-transgender episodes. Authors conducted two focus groups to qualitatively explore resilience strategies, reporting that resilience represented a fundamental coping strategy to cope with anti-transgender discrimination, and that, similar to the findings observed by Singh et al. [21,22], it dealt with both individual traits, such as identity affirmation and self-acceptance, and social characteristics, such as the capacity to make use of peer group as a source of support. 

### 1.4. The Current Study

The present study aimed at applying part of the PMF to a sample of Italian TGNC adults, while it replicated a part of a model already tested by Breslow et al. [5], but without statistical significance. Indeed, in the study by Breslow et al. [5] only stigma awareness as a proximal stressor and collective action as a group-level coping mechanism shed light on psychological processes through which anti-transgender discrimination affects health. Surprisingly, internalized transphobia as a proximal stressor and resilience as an individual-level coping mechanism did not contribute to the model. As previously said, it is possible that this part of the model was not significant due to the use of a measure not specifically created for assessing internalized transphobia (both horizontal and vertical dimensions) in the TGNC population. Summarizing, we were interested in: (1) replicating a moderated-mediation model that did not results significant in the study by Breslow et al. [5]; (2) taking into account both vertical and horizontal internalized transphobia [15]; and (3) applying part of the PMF to a sample of Italian TGNC people.

Thus, based on the MST [7,41], we hypothesized that anti-transgender discrimination and internalized transphobia would be positively associated with negative mental health outcomes, assessed through anxiety and depression measures, and that resilience would be negatively associated with mental health problems (Hypothesis 1). Then, informed by the PMF [10], we hypothesized that both vertical and horizontal internalized transphobia (i.e., shame and alienation) would act as a mediator between anti-transgender discrimination and mental health (Hypothesis 2). Furthermore, on the basis of the PMF as well as on recent studies focusing on resilience in the TGNC population [20,21,22,23,42], we hypothesized that resilience would buffer the relationship between anti-transgender discrimination and mental health, as well as between vertical and horizontal internalized transphobia (i.e., shame and alienation) and mental health (Hypothesis 3). Specifically, we hypothesized that the effect of anti-transgender discrimination and internalized transphobia on mental health would be weaker when individuals display higher resilience levels. Finally, we tested whether the indirect effect of anti-transgender discrimination on mental health through shame or alienation was moderated by resilience (Hypothesis 4). 

**Figure 1:**
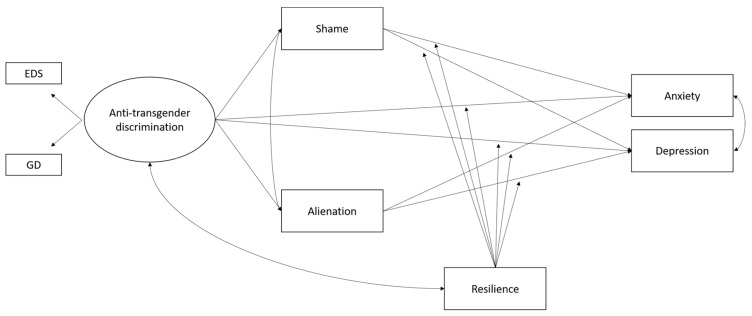
The hypothesized moderated mediation model. Note: EDS = everyday discrimination; GD = general discrimination. For simplification reasons, control variables and covariations between shame and resilience, and alienation and resilience were not reported in the figure.

The hypothesized moderated-mediation model is depicted in Figure 1.

## 2. Materials and Methods

### 2.1. Participants

Data from 149 Italian TGNC individuals were analyzed in the current study. Participants ranged in age from 18 to 63 years old (M = 33.18, SD = 10.96). Seventy-five participants identified as male-to-female (MtF) and 74 as FtM. Participants could participate in the online survey only if they self-identified with a TGNC identity, were at least 18-years-old (the Italian age of consent), and had lived in Italy for at least 10 years. Demographic characteristics are reported in Table 1.

### 2.2. Procedures

This study used a cross-sectional online survey administered through Qualtrics [43]. By clicking on a link, participants were directed to the first page of the survey containing the informed consent of the study where information about the researchers and their e-mail addresses and telephone numbers were provided. Before starting the survey and after reading what would be asked and what risks and benefits the survey entailed, participants had to give their consent by clicking on the button “I give consent to participate in the survey”. Subsequently, participants were screened for their eligibility. Individuals participated in a lottery where 10 participants were randomly selected to receive 100 €. At the end of the survey, we asked participants to provide their personal email on a voluntary basis, guaranteeing that all personal information would be disassociated from data they reported. The system was set up to allow answering the survey only once and gave participants the opportunity to save their responses and answer questionnaires within a week’s time from their first access.

The survey was launched on social media, such as Facebook, and all Italian associations promoting TGNC rights were contacted and invited to disseminate the survey to their contacts, activating a snowball sampling recruitment procedure.

Privacy was guaranteed in accordance with Italian law 196/2003. Indeed, data were protected by a secure gateway accessible only to the Principal Investigator (PI), who removed all IP addresses and saved the emails of participants who decided to take part in the lottery on a separate sheet. Only after these procedures were performed, the PI shared the dataset with other scholars. The study was designed to respect of all the principles of the Declaration of Helsinki on Ethical Principles for Medical Research Involving Human Subjects.

### 2.3. Measures

#### 2.3.1. Socio-Demographic Characteristics

Socio-demographic variables included sex assigned at birth, gender identity (male, female, and other with specification required), age, level of education (0 = high school or less; 1 = college or more), monthly income, in a romantic relationship (0 = no; 1 = yes), size of community (1 = urban; 2 = suburban; 3 = rural), belonging to a trans association (0 = no; 1 = yes), and religious education (0 = no; 1 = yes).

#### 2.3.2. Anti-Transgender Discrimination

We used two measures to assess anti-transgender discrimination. We assessed general discrimination through nine items asking participants whether they had been fired, rejected when they tried to rent an apartment, evicted, robbed, experienced trouble in finding a job, experienced problems when accessing health services, verbally, physically, and sexually abused. This scale was previously used in an Italian study on TGNC population [8]. Respondents indicated to what extent they suffered from each type of discrimination on a five-point Likert scale, from 0 (never) to 4 (very often). Each item was directly linked to TGNC identity, asking “Considering your transgender identity or expression as the cause, how often have you experienced the following situation?” The internal consistency reliability of the measure was α = 0.77.

We also assessed everyday discrimination through the Everyday Discrimination Scale (EDS). This scale was originally developed by Williams et al. [44] to measure everyday discrimination episodes in diverse ethnic groups. Then, it was adapted to sexual minority groups by Meyer et al. [45]. A recent Italian study [8] adapted EDS to Italian TGNC population. This scale evaluates the frequency of nine types of everyday discrimination episodes: being treated with less courtesy, being treated with less respect, receiving poorer services, being treated as not smart, perceiving that people act as if they are afraid of you, perceiving that people act as if you are dishonest, perceiving that people act as if they are better than you, being called names or insulted, and being threatened or harassed. The scale was adapted to the TGNC population, asking: “In your day-to-day life how often have any of the following things happened to you due to your gender identity or expression?” Respondents indicated to what extent they suffered from each everyday discrimination on a four-point Likert scale, from 0 (never) to 4 (often). The internal consistency reliability of the measure was α = 0.91.

#### 2.3.3. Internalized Transphobia

To assess both vertical and horizontal internalized transphobia we used two subscales of the Transgender Identity Survey (TIS) [17,46]. Specifically, to assess the vertical internalized transphobia we administered the subscale Shame, comprising eight items assessing self-hatred and shame towards one’s own TGNC identity (e.g., “Being transgender makes me feel like a freak”). To assess the horizontal internalized transphobia we administered the subscale Alienation, comprising three items assessing alienating feelings towards other TGNC people (e.g., “I feel isolated and separate from other transgender people”). The response options ranged from 1 (strongly disagree) to 7 (strongly agree). Higher scores on both subscales indicate higher levels of internalized transphobia. The internal consistency reliability was α = 0.89 for Shame and α = 0.82 for Alienation.

#### 2.3.4. Depression

Depression was assessed through the Center for Epidemiologic Studies Depression Scale (CES-D) [47,48], a 20-item measure evaluating depressive symptoms during the previous week on a four-point Likert scale, from 0 (rarely or none of the time—less than 1 day) to 3 (all of the time—5–7 days). An example item is “I was bothered by things that usually don’t bother me.” The internal consistency reliability was α = 0.94. In the TGNC population, a score of 16 was used as the cut-off point for high depressive symptoms [49]. In the current sample, 49 (65.3%) MtF and 45 (60.8%) FtM TGNC individuals met the clinical cut-off. No statistical difference between two groups was detected (*p* = 0.568)

#### 2.3.5. Anxiety

Anxiety was assessed through the Beck Anxiety Inventory (BAI) [50,51], a 21-item measure evaluating anxious symptoms (such as fear of losing control or difficulty in breathing) during the previous month on a four-point Likert scale, from 0 (not at all) to 3 (severely). The internal consistency reliability was α = 0.95. In the Italian normative sample, a score of 13 was used as the cut-off point for high anxiety symptoms. In the current sample, 32 (42.7%) MtF and 38 (51.4%) FtM TGNC individuals met the clinical cut-off. No statistical difference between two groups was detected (*p* = 0.289).

#### 2.3.6. Resilience

Resilience was assessed through the Resilience Scale (RS) [52,53], a 10-item measure evaluating resilience on a seven-point Likert scale, from 1 (strongly disagree) to 7 (strongly agree). In this scale, resilience was conceptualized as a personal characteristic buffering the negative effects of stress and promoting adjustment. An example item is “When I’m in a difficult situation, I can usually find my way out of it.” The internal consistency reliability was α = 0.90.

### 2.4. Preliminary and Statistical Analyses

Preliminary analyses mainly concerned the handling of missing data and outliers. Missing data were treated through a multiple imputation procedure [54], using Amelia II package for R. Moreover, as suggested by Tabachnick and Fidell [55], univariate outliers were searched through a standardized score greater than 3.29 or smaller than −3.29. Additionally, multivariate outliers were searched through the Mahalanobis distance. No participants satisfied criteria to be removed.

All the study’s hypotheses were tested using structural equation modeling (SEM) in Mplus 7.2 [56]. Based on values of skewness and kurtosis showing no substantial deviations of variables from normal distribution, analyses were performed by using the maximum likelihood (ML) estimator. One latent factor of anti-transgender discrimination indicated by observed measures of general discrimination and everyday discrimination was estimated as part of the main structural equation model. On the contrary, we decided to not create a unique latent variable measuring mental health and to maintain anxiety and depression as separate variables because, as suggested by Clark and Watson [57] in their tripartite model of anxiety and depression, general distress or negative affect is the only characteristic that both shared. On the contrary, the physiological hyperarousal was considered to be specific to anxiety and the absence of positive affect was specific to depression. On this basis, we were interested in exploring whether anti-transgender discrimination and internalized transphobia would affect different dimensions of mental health.

Several confounding variables were considered in the study, as they are thought to influence both stressors and mental health [3,6,41]: sex assigned at birth, age, monthly income, actual romantic status, size of community, belonging to a trans association, and religious education. However, since size of community, level of education, and religious education showed no statistically significant correlations with any of the variables included in the study, they were excluded from the analyses.

Analyses were performed in a series of steps. To address our first hypothesis, we initially tested the main effects of anti-transgender discrimination, internalized transphobia, and resilience on mental health problems (anxiety and depression); then, we examined whether internalized transphobia mediated the relationship between anti-transgender discrimination and mental health problems (Hypothesis 2). Multiple fit indices were used to evaluate model fit: chi-square likelihood ratio statistic (*χ*^2^), comparative fit index (CFI), and the root mean square error of approximation (RMSEA) with associated 90% confidence intervals. According to the indication provided by Hu and Bentler [58], acceptable model fit was defined by the following criteria: non-significant of *χ*^2^ value, CFI ≥ 0.95, and RMSEA ≤ 0.08. Next, we tested the moderating effect of resilience in the relationships between anti-transgender discrimination and mental health, as well as between internalized transphobia and mental health (Hypothesis 3). Interactions that did not significantly predict any of the observed outcome variables were trimmed for parsimony in the final model; significant conditional effects were probed by using the pick-a-point approach conditioned on values of the moderator corresponding to the sample mean and a standard deviation below and above the mean [59]. As a final step, in order to provide evidence of moderation of the indirect effect (Hypothesis 4), we calculated the indices of moderated mediation for each of the hypothesized moderated paths, as recommended by Hayes [60]. Both mediation [61] and moderated mediation [60] were tested by using bias-corrected bootstrap confidence intervals based on 5000 resamples, as an indicator of effect size. Confidence intervals that do not contain zero indicate a significant indirect effect via the specific mediator.

## 3. Results

### 3.1. Descriptive Statistics and Bivariate Correlations

Means, standard deviations, and bivariate correlations between all variables are shown in Table 2. The results highlighted a positive correlation between general and everyday discrimination. 

Both measures of anti-transgender discrimination were positively associated with alienation and depression, and negatively associated with depression and resilience. Contrary to general discrimination, everyday discrimination was also positively associated with shame and anxiety.

Shame and alienation were positively correlated with both anxiety and depression. A negative association was found between resilience and shame, as well as between resilience and alienation. Similarly, a negative association was also found between resilience and depression, and between resilience and anxiety.

Among the control variables, age was significantly and negatively associated with shame and anxiety, while being in a romantic relationship was associated with low alienation and with both low general and everyday discrimination. Resilience correlated positively with being in a romantic relationship and belonging to a trans association. Size of community, level of education, and religious education did not correlate with any variables in the study, so they were removed from further analyses.

### 3.2. Associations between Minority Stressors, Resilience, and Mental Health

**Figure 2:**
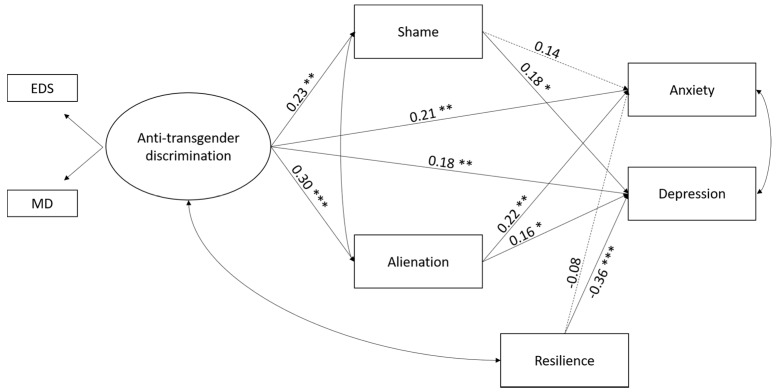
Results from the structural equation modeling of the hypothesized moderated mediation model. Note: EDS = everyday discrimination; GD = general discrimination. Standardized path coefficients are reported. Dashed lines indicate non-significant paths. For simplification reasons, control variables and covariations between shame and resilience, and alienation and resilience, were not reported in the figure. *** *p* < 0.001; ** *p* < 0.01; * *p* < 0.05.

As shown in Figure 2 and regarding Hypothesis 1, anti-transgender discrimination was positively associated with anxiety and depression. High alienation was associated with both anxiety and depression, whereas high shame predicted depression only. Resilience was negatively associated with depression, but not with anxiety. The model showed an acceptable fit to the data, as follows: χ^2^ = 14.64, *p* = 0.15, CFI = 0.99, RMSEA = 0.05 with 90% C.I. [0.00, 0.11].

#### Control Variables

Being in a romantic relationship was associated with high resilience and low anti-transgender discrimination, whereas belonging to a trans association was associated with low shame and high resilience. Age was negatively associated with shame.

### 3.3. Internalized Transphobia as a Mediator and the Moderating Role of Resilience

Regarding Hypothesis 2, we found that alienation mediated the relationship between anti-transgender discrimination and both anxiety and depression, bs = 0.91 and 0.60, 95% C.I.s [0.17, 2.28] and [0.08, 1.56], respectively, whereas shame operated as a mediator of the relationship between anti-transgender discrimination and depression, b = 0.55, 95% C.I. [0.08, 1.55].

**Figure 3:**
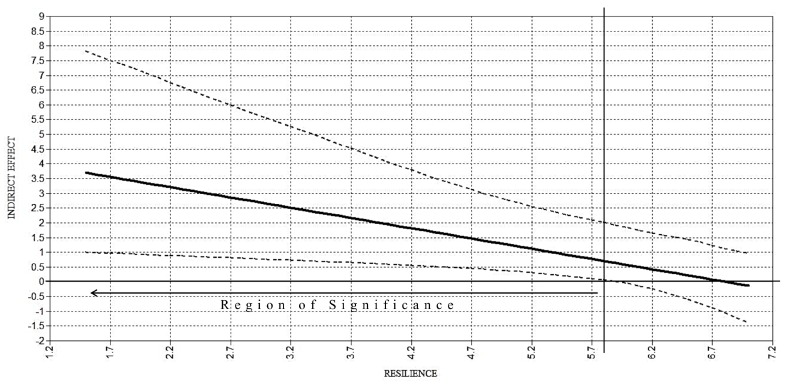
Plot of the conditional indirect effect of anti-transgender discrimination on anxiety through alienation. Note: dashed lines indicate 95% confidence bands. The horizontal line denotes an indirect effect of zero. The vertical line represents the boundary of the region of significance.

The moderation analyses highlighted only one significant interaction between alienation and resilience on anxiety, β = −0.53, *p* < 0.001, partially confirming Hypothesis 3. Following the pick-a-point procedure, the positive effect of alienation on anxiety was found to be significantly stronger in individuals with low resilience, b = 3.49, 95% C.I. [1.52, 5.23], and moderate resilience, b = 1.93, 95% C.I. [0.47, 3.49]. Among those highly resilient, alienation had no significant effect on anxiety, b = 0.37, 95% C.I. [−1.46, 2.43]. Additionally, the estimation of the moderated mediation indices revealed only one significant moderated indirect effect, ω = −0.70, 95% C.I. [−1.60, −0.14], indicating that the indirect relation of anti-transgender discrimination with anxiety through alienation was conditional on low and moderate levels of resilience, bs = 1.73 and 0.96, 95% C.I.s [0.54, 3.60] and [0.22, 2.33], respectively, partially confirming Hypothesis 4 (Figure 3).

## 4. Discussion

Informed by the PMF as an extension of the MST [10], the current study explored the role of internalized transphobia as a mediator between anti-transgender discrimination and mental health. Furthermore, informed by studies focusing on resilience in the TGNC population [20,21,22,23,42], this study also explored resilience as a buffering dimension of the relationship between anti-transgender discrimination and mental health, as well as between internalized transphobia and mental health. Yet, contrary to Breslow et al. [5], we found partial support for this model. Thus, our results shed light on clinical practice and psycho-social interventions, meeting the need of structuring effective interventions to reduce stigma and stress caused by interpersonal and individual stigma [62]. 

In support of the first hypothesis of this study, we found that anti-transgender discrimination and internalized transphobia were positively associated with negative mental health outcomes, and that resilience was negatively associated with mental health problems. These results are consistent with previous findings on MST among TGNC people [3,4,9,19], as well as with those highlighting the existence of adaptive strategies able to buffer the detrimental effect of minority stress on health [20,21,22,23,42]. Specifically, we found that horizontal internalized transphobia (i.e., alienation) was positively associated with both anxiety and depression, while vertical internalized transphobia (i.e., shame) only with depression. This result is interesting as it shows a different impact that internalized transphobia might have on mental health on the basis of its dimensionality, as hypothesized. Based on the tripartite model of anxiety and depression [57], there is evidence that the physiological hyperarousal is specific to anxiety and the absence of positive affect is specific to depression. This leads us to postulate that anxiety has more to do with fearfulness, while depression with a sense of hopelessness or loss of self-esteem. From our results, it seems that vertical internalized transphobia especially affects depressive mood, probably because feeling shame towards one’s own TGNC identity and negative self-judgment is strictly connected with intensive negative feelings which lead to perceive oneself as devalued and unworthy. In contrast, horizontal internalized transphobia also affects anxiety, probably because alienating feelings towards other TGNC people and isolation ends up producing vulnerability and weakness with respect to the environmental difficulties which, in turn, can cause hypercontrol and hyperarousal. Notwithstanding these interpretative hypotheses, these results ought to be thoroughly and qualitatively investigated to better understand how psychological and social processes lead internalized transphobia to affect different health dimensions.

Regarding the second hypothesis, or rather the mediating role of vertical and horizontal internalized transphobia of the relationship between anti-transgender discrimination and mental health, our findings provided partial support. First, regarding the predictors within the mediational model, anti-transgender discrimination was positively associated with both vertical and horizontal internalized transphobia. This is consistent with previous studies which found that prejudice events are positively associated with internalized stigma [63]. Second, both vertical and horizontal internalized transphobia mediated the relationship between anti-transgender discrimination and depression. Differently, our results indicated that horizontal (i.e., alienation)—but not vertical (i.e., shame)—internalized transphobia mediated the relationship between anti-transgender discrimination and anxiety. These findings are generally in line with prior research in which internalized stigma mediated the relation between discrimination and mental health in samples of lesbian women and gay men [64]. Furthermore, these findings are consistent with those related to our first hypothesis, inasmuch as the indirect effect of anti-transgender discrimination on anxiety and depression depends on the specific dimensionality of internalized transphobia.

In support of the third hypothesis, or rather the role of resilience as a moderator between anti-transgender discrimination and mental health, and between vertical and horizontal internalized transphobia and mental health, our results indicated that resilience only significantly moderated the relationship between horizontal internalized transphobia (i.e., alienation) and anxiety. Specifically, only participants with high resilience levels seemed able in resisting the negative effects of alienating feelings towards other TGNC people on anxiety. This is consistent with studies highlighting the protective role of resilience in sexual and gender minority people [20,21,22,23,42]. This interaction indicated a case of moderated mediation, in support of our fourth hypothesis. Indeed, our results highlighted the importance of resilience in moderating the indirect effect of anti-transgender discrimination on anxiety through alienation. That is, alienation did not mediate the relation between anti-transgender discrimination and anxiety in highly-resilient individuals, pointing to the protective role of resilience in coping with the negative effects of stigma on mental health.

### 4.1. Limitations and Suggestions for Future Research

Results of the current study must be read in light of important limitations which might affect their generalizability to the general Italian TGNC population. First, the cross-sectional design of this study does not allow us to make inferences about the directional nature of the investigated relationships. Future longitudinal studies are needed to discern the cause-effect relationships between stigma, internalized transphobia, resilience, and mental health. 

Moreover, looking at the sample composition, the present study analyzed data from individuals identifying only with binary gender identities and we cannot extend our findings to individuals identifying with non-binary identities, such as genderqueer or gender fluid. The absence of non-binary identities might be due to socio-cultural issues, as the trend to self-identify within a binary identity seems to still be prevalent in Italy [65,66,67]. Similarly, since participants were almost exclusively Caucasian, we were not able to assess the role of ethnicity on the associations between stigma, internalized transphobia, resilience, and mental health. This is a typical limitation of Italian TGNC people samples [8]. Indeed, non-Caucasian TGNC individuals living in Italy are mainly South American, a highly vulnerable and not socially-integrated population [68]. This makes their recruitment difficult to achieve. This limitation should bring Italian researchers to use different forms of recruitment, crossing the barriers to accessibility.

Furthermore, the resilience scale used in the current study assesses above all else the individual traits of the construct. Future studies should also consider to quantitatively assess the social characteristics of resilience in TGNC individuals, capturing all nuances that previous studies [21,22,40] have qualitatively observed. This might provide a more complete understanding of resilience in TGNC individuals.

Finally, the use of self-report measures as indicators of anxiety and depression (i.e., BAI and CESD) represents another important limitation as they are explicit measures. To overcome this limitation, future research should consider administering semi-structured clinical interviews as implicit measures providing more accurate data. 

### 4.2. Implications for Clinical Practice

The findings of the current study might have helpful suggestions for clinical practice with TGNC people despite limitations. Indeed, MST and its extension (i.e., PMF) were developed to deeply understand psychological and social processes affecting the health of sexual minorities and were subsequently extended to TGNC individuals. 

As suggested by White Hughto et al. [62], stigma affects TGNC mental health at multiple levels, such as the structural, interpersonal, and individual. In the current study, we did not address anti-transgender stigma at the structural-level, not assessing the impact that discriminatory policies has on health and on access to resources. On the contrary, we addressed stigma at an interpersonal-level (e.g., enacted forms of stigma, such as violence) and individual-level (i.e., internalized transphobia). Thus, on the basis of our results, we can report suggestions related to effective interventions whose scope is reducing stress and its negative impact on mental health on the abovementioned levels. 

Considering the interpersonal-level, among effective psychological interventions in reducing stigma and health risk are those helping TGNC people becoming acquainted, sharing strategies to increase resilience [69]. An example might be an empowerment-based training group conducted by field experts and addressed to TGNC individuals. For instance, a recent Italian study [40], in presenting a training module to enhance resilience levels in TGNC youths experiencing transphobic violence, emphasized the role of the peer group in increasing resilience and wellbeing, in line with the international scientific literature [2,3]. It seems to us that this suggestion is particularly in line with the results related to the role of horizontal internalized transphobia as a mediator between anti-transgender discrimination and negative mental health outcomes. Indeed, contact with other TGNC people might alleviate alienating feelings facilitating the use of strategies to cope with isolation and thus increasing wellbeing. Likewise, it might mitigate feelings of shame towards one’s own TGNC identity, sharing them with peers and feeling less alone. Furthermore, these interventions ought to also be addressed to individuals who may be in a position to limit access to resources for the TGNC population, such as family members, peers, and healthcare professionals [70]. For instance, family support groups might help non-TGNC family members to develop a humanizing view of their TGNC family members or to cope with the societal stigma that families with a TGNC member may experience. Similarly, healthcare professionals should be trained to recognize and break down healthcare barriers that TGNC individuals often encounter and to reshape their implicit gender biases that may influence their healthcare practices. To this end, contact between healthcare professionals and TGNC individuals might be beneficial in eliminating biases and discomfort [62].

Considering the individual-level, instead, interventions aimed at reducing stigma and its negative impact on mental health are typically psychological-clinical interventions. For instance, among their objectives, and far from a pathologizing perspective [71], counseling or individual psychotherapy could alleviate negative effects stemming from internalized transphobia on self-perception. On the basis of our study, counselors and therapists should work on feelings of shame towards one’s own TGNC identity, helping clients to reshape negative emotions associated with stigmatizing experiences and assist them in developing a self-image freer from binary gender. In the same vein, they should also consider group approach as a valid alternative to individual work. Indeed, by its nature, the group encourages mirroring processes that facilitate reshaping self-image in an innovative and potentially creative way [72], increasing self-empowerment processes and resilience strategies [40].

## 5. Conclusions

Notwithstanding the limitations, the current study provides further support for the application of MST to the TGNC population [6,7], as well as its recent extensions in terms of pattern of mediation (i.e., PMF) [10]. Within the TGNC population, Breslow et al. [5] found support only for the PMF part that considers stigma awareness as a mediator and group-level coping strategies as a moderator. On the contrary, they did not find statistical significance for the role of internalized transphobia as a mediator and resilience as a moderator. In our study, instead, probably due to the use of a measure explicitly created to assess internalized transphobia in the TGNC population, we provide further evidence on the effectiveness of the PMF, while also differentiating the role of the two dimensions of internalized transphobia: shame and alienation. The main result of the current study was related the moderated-mediation model, indicating that alienation only mediates the relationship between anti-transgender discrimination and mental health, and that this is true solely if resilience levels are low or moderate. This finding fully satisfies the PMF, shedding light on psychological processes that lead both anti-transgender discrimination to affect mental health and protective factors to alleviate the negative effect of stigma on mental health.

## Figures and Tables

**Table 1:** Socio-demographic characteristics of participants (*N* = 149).

Characteristics	Total (*N* = 149) *N* (%) or M ± SD	Male to Female (*N* = 75) *N* (%) or M ± SD	Female to Male (*N* = 74) *N* (%) or M ± SD	*p* Value
Age	33.18 ± 10.96	37.21 ± 12.24	29.22 ± 7.77	<0.001
Ethnicity				0.368
Caucasian	147 (98)	74 (98.7)	73 (98.6)	
African	1 (0.7)	1 (1.3)	–	
Latino	1 (0.7)	–	1 (1.4)	
Education				0.552
≤High school	106 (71.1)	55 (73.3)	51 (68.9)	
≥College	43 (28.9)	20 (26.7)	23 (31.1)	
Monthly income (€)				0.492
No income	59 (39.6)	30 (40)	29 (39.2)	
<600	24 (16.1)	12 (16)	12 (16.2)	
600–999	31 (20.8)	12 (16)	19 (25.7)	
1000–1999	20 (13.4)	10 (13.3)	10 (13.5)	
2000>	15 (10.1)	11 (14.6)	4 (5.4)	
Marital status				0.004
Unmarried	127 (85.2)	56 (74.7)	71 (95.9)	
Married	9 (6)	7 (9.3)	2 (2.7)	
Widowed	2 (1.3)	1 (1.3)	1 (1.4)	
Divorced	3 (2)	3 (4)	–	
Separated	8 (5.4)	8 (10.7)	–	
Community size				0.768
Urban	111 (74.5)	55 (73.3)	56 (75.7)	
Suburban	19 (12.8)	9 (12)	10 (13.5)	
Rural	19 (12.8)	11 (14.7)	8 (10.8)	
Trans association				0.788
Yes	58 (38.9)	30 (40)	28 (37.8)	
No	91 (61.1)	45 (60)	46 (62.2)	
Religious education				0.247
Yes	109 (73.2)	58 (77.3)	51 (68.9)	
No	40 (26.8)	17 (22.7)	23 (31.1)	

Note: M = mean; SD = standard deviation; Group differences in age were assessed through Student’s *t* test; Group differences in all other characteristics were assessed through the *χ*^2^ test.

**Table 2:** Descriptive statistics and bivariate correlations between minority stressors, mental health, resilience, and socio-demographic characteristics.

*Main Variables*	**1**	**2**	**3**	**4**	**5**	**6**	**7**	**8**	**9**	**10**	**11**	**12**	**13**	**14**	**15**	**Mean**	**SD**
1. General discrimination	1															12.35	2.47
2. Everyday discrimination	0.65 ***	1														1.92	0.73
3. Shame	0.10	0.20 *	1													3.39	1.57
4. Alienation	0.24 ***	0.33 ***	0.37 ***	1												3.42	1.78
5. Anxiety	0.12	0.33 ***	0.32 ***	0.33 ***	1											16.36	14.89
6. Depression	0.19 *	0.40 ***	0.43 ***	0.38 ***	0.69 ***	1										22.90	14.05
7. Resilience	−0.23 ***	−0.34 ***	−0.42 ***	−0.26 ***	−0.29 ***	−0.55 ***	1									5.44	1.11
*Control variables*																	
8. Gender (MtF)	0.12	−0.20*	0.12	−0.16	0.10	0.03	−0.03	1									
9. Age	0.02	0.01	−0.24 ***	0.01	−0.25 ***	−0.14	0.15	−0.36 ***	1								
10. Education (≤High school)	−0.09	−0.09	0.01	0.11	−0.04	−0.01	−0.01	0.06	0.11	1							
11. Monthly income	−0.22 ***	−0.15	−0.09	−0.03	−0.20 *	−0.21 **	0.11	−03	0.34 ***	0.12	1						
12. Being in a relationship	0.24 ***	0.23 **	0.11	−0.21 ***	−0.03	−0.14	0.20 *	0.32 ***	−0.13	−0.02	0.01	1					
13. Community size	0.06	0.03	0.04	0.01	−0.01	0.04	0.05	−0.05	0.01	−0.05	−0.09	0.05	1				
14. Trans association	0.03	−0.08	0.29 ***	0.10	0.03	−0.11	0.20 *	−0.02	0.02	0.05	−0.01	−0.13	0.05	1			
15. Religious education	−0.04	−0.07	−0.01	0.03	−0.07	−0.09	0.05	0.10	−0.14	−0.04	−0.10	−0.04	−0.16 *	0.14	1		

Note: SD = standard deviation; *** *p* < 0.001; ** *p* < 0.01; * *p* < 0.05.
